# Sequential CDK4/6 inhibition in bone-only metastatic HR+/HER2- breast cancer: a case of prolonged disease control with abemaciclib after clinical progression on palbociclib-based therapy

**DOI:** 10.3389/fmed.2026.1775724

**Published:** 2026-05-08

**Authors:** Xuejiao Peng, Weiqin Chang

**Affiliations:** Department of General Surgery, The Second Hospital of Jilin University, Changchun, Jilin, China

**Keywords:** abemaciclib, bone-only metastasis, case report, CDK4/6 inhibitor switch, endocrine resistance, palbociclib

## Abstract

**Background:**

The efficacy of sequential cyclin-dependent kinase (CDK4/6) inhibition after clinical progression on a prior CDK4/6 inhibitor remains controversial, particularly in patients with bone-only hormone receptor-positive/HER2-negative (HR+/HER2-) metastatic breast cancer (MBC), for whom evidence remains limited. Herein, we describe prolonged clinical benefit with abemaciclib after clinical progression during palbociclib-based therapy in this setting.

**Case report:**

A 59-year-old woman with stage IIIA HR+/HER2- breast cancer underwent modified radical mastectomy in 2016, followed by adjuvant AC-P (doxorubicin hydrochloride 70 mg, cyclophosphamide 800 mg, and paclitaxel liposomes 180 mg) chemotherapy, radiotherapy, and letrozole maintenance. Two years later, she developed an isolated scapular metastasis with secondary endocrine resistance. Since palbociclib was not yet covered by insurance, she received an initial chidamide + exemestane therapy, which proved ineffective. Using second-line palbociclib combined with fulvestrant and zoledronic acid therapy, disease stabilization was achieved for up to 12 months. Subsequently, CA15-3 marker levels increased, accompanied by progressive osteolytic bone destruction and bone pain. When abemaciclib was included in China’s national reimbursement list in January 2022, the regimen was switched to abemaciclib + fulvestrant + zoledronic acid. CA15-3 levels normalized within 3 months; moreover, serial imaging revealed no new lesions. As of September 2023, the patient has remained progression-free for >20 months and experienced only manageable grade 2 diarrhea.

**Conclusion:**

This case suggests that sequential CDK4/6 inhibition could help achieve >20 months of progression-free disease control in a patient with bone-only HR+/HER2- MBC after clinical progression during prior palbociclib-based therapy and underscored the need for biomarker-guided strategies in this distinct population.

## 1 Introduction

Breast cancer is a commonly diagnosed malignancy, accounting for 25% of cancer cases in women worldwide. Approximately 70% of breast cancers are hormone receptor positive (HR+). Cyclin-dependent kinase 4/6 (CDK4/6) inhibitors are effective BC therapies and, in combination with endocrine therapy, form the first-line standard of care for managing patients with HR+, HER2- advanced breast cancer (1). The availability of CDK4/6 inhibitors has greatly improved prognosis of advanced HR+/HER2- breast cancer (2). Three large randomized phase III trials, the PALOMA-2, MONALEESA-2, and MONARCH-3, investigated CDK4/6 inhibitors palbociclib, ribociclib, and abemaciclib, respectively, in combination with nonsteroidal aromatase inhibitors for the first-line treatment of postmenopausal HR+ and HER2-advanced breast cancer (3–5). All three CDK4/6 inhibitors substantially prolonged progression-free survival (PFS); furthermore, the hazard ratios for PFS were nearly identical. CDK4/6 inhibitors have rapidly become the standard first-line regimen recommended by global guidelines. In the past 3 years, the greatest progress has been made in adjuvant therapy, with two landmark trials, the MONARCH-E and NATALEE (6, 7). They indicated that abemaciclib and ribociclib significantly reduced invasive disease-free survival events in patients with high-risk, early-stage HR+ breast cancers, opening a new chapter in the “cure-enhanced” treatment landscape. Dalpiciclib was approved by the National Medical Products Administration based on the DAWNA-1/2 trials, providing a new, affordable, and accessible option for treating patients with localized cancer.

CDK4/6 inhibitors combined with endocrine therapy show promising efficacy; however, drug resistance remains a challenge. No standard subsequent-line regimen has been defined for disease progression. Real-world data show that when patients switch directly to a second CDK4/6 inhibitor after progression on the first agent, the median PFS achieved with the second agent is only approximately 5–8 months (8). Herein, we present a case of bone-only HR+/HER2- metastatic breast cancer that achieved more than 20 months of progression-free disease control with sequential CDK4/6 inhibition after clinical progression during palbociclib-based therapy, switching from palbociclib to abemaciclib in combination with fulvestrant and zoledronic acid. Together with the current literature, this case adds clinical context to the ongoing discussion regarding sequential CDK4/6 inhibition and the need for more precise patient stratification.

## 2 Case presentation

**FIGURE 1:**
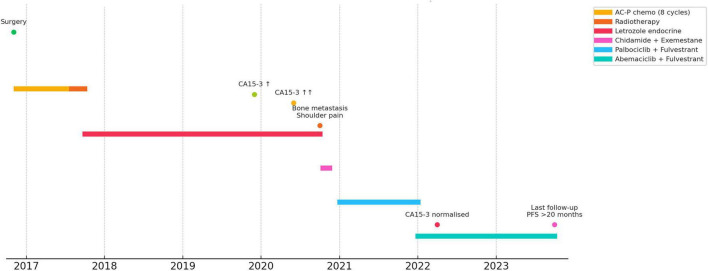
Integrated treatment timeline and key clinical events in the case of a patient with HR+/HER2- metastatic breast cancer.

A 59-year-old woman underwent a modified radical mastectomy on November 3, 2016, for left-sided invasive ductal carcinoma. Her treatment timeline is illustrated in Figure 1. At disease recurrence, the patient’s ECOG performance status was 0. There were no remarkable past medical conditions or known drug allergies. No family history of breast or ovarian cancer existed among her first-degree relatives. The patient did not smoke or consume alcohol. She enjoyed strong family support and exhibited excellent treatment compliance. Histology revealed a 2.1 cm, grade II tumor with metastases in four of the 29 dissected axillary lymph nodes; pathologic staging was pT2N2M0 (stage IIIA). Immunohistochemistry showed ER (++), PR (+), HER2 (1+), p53 (focal +), and a Ki-67 index of approximately 30 %.

The patient received eight cycles of chemotherapy with the AC-P regimen (doxorubicin hydrochloride 70 mg, cyclophosphamide 800 mg, and paclitaxel liposomes 180 mg), radiotherapy to the chest wall and regional lymph nodes (DT 50 Gy in 25 fractions), and letrozole as adjuvant endocrine therapy. A review in December 2019 showed mild elevation of the following tumor markers: CA15-3:33.95 U/mL (reference value: 0–25 U/mL), asymptomatic, and no imaging abnormalities. Although CA15-3 levels further increased to 66.25 U/mL, the patient remained asymptomatic, and imaging findings were unremarkable. The use of a single tumor marker to detect early recurrence has limited clinical utility (9). According to the 2018 edition of the Chinese Expert Consensus on Clinical Diagnosis and Treatment of Advanced Breast Cancer (10), the persistent elevation of tumor markers during the treatment of advanced breast cancer may be an early manifestation of tumor progression, which should be combined with imaging examinations at the same time to clarify the diagnosis and determine whether a change in treatment is warranted. Simple elevation of tumor markers cannot be used as a basis for changing the treatment regimen and dynamic observation and reexamination within 1–2 months are recommended. Since the patient had a purely elevated tumor marker without symptoms or imaging changes, we chose to review the findings after 2 months.

**FIGURE 2:**
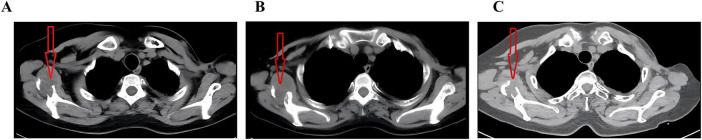
CT images showing metastatic lesions. **(A)** Initial CT scan showing a metastatic lesion in the right scapula region (arrow). **(B)** Follow-up CT scan showing mild enlargement of the lesion after treatment (arrow). **(C)** CT scan after sequential abemaciclib treatment showing no significant progression of the metastatic lesion (arrow).

On October 1, 2020, the patient presented with pain and discomfort in the right shoulder, which became obvious after activity. The CA15-3 level had risen to 99.25 U/mL, and CT revealed osteolytic destruction of the right scapula (Figure 2A). Positron emission tomography-CT showed bone destruction in the right scapula, accompanied by a soft tissue mass. The remaining organs (the brain, lungs, and liver) showed no abnormalities. Diagnosis: The patient was diagnosed with bone metastasis after postoperative chemoradiotherapy for advanced-stage invasive ductal carcinoma of the left breast. The recurrence occurred after approximately 2 years of adjuvant endocrine therapy and was considered consistent with secondary endocrine resistance. Since she had only bone metastasis without visceral metastasis or visceral crisis, endocrine therapy was preferred according to the national and international guidelines (10–12). The 2020 edition of the CSCO guidelines for breast cancer diagnosis and treatment recommends steroidal AIs + HDAC inhibitors or fulvestrant + CDK4/6 inhibitors for treating patients whose cancer progressed after NSAI treatment. Given the patient’s economic situation and fact that CDK4/6 inhibitors were not covered by health insurance at that time, a combination of chidamide, exemestane, and zoledronic acid was chosen. However, the patient continued to experience shoulder and back pain, and tumor marker levels remained elevated. In addition, she developed significant adverse effects, including nausea, vomiting, and electrolyte disturbances. Therefore, in January 2021, the patient was switched to a fulvestrant, palbociclib (125 mg; d1–21, q28d), and zoledronic acid regimen. After the switch of medication, the patient experienced marked relief of shoulder and back pain, together with a transient decline in serum CA15-3 levels. During subsequent follow-up, however, CA15-3 levels increased again, imaging showed interval enlargement of the osteolytic destruction (Figure 2B), and the shoulder and back pain gradually recurred. The patient became increasingly concerned because of the persistently rising tumor markers, progressive osteolytic changes on imaging, and recurrent shoulder and back pain.

**FIGURE 3:**
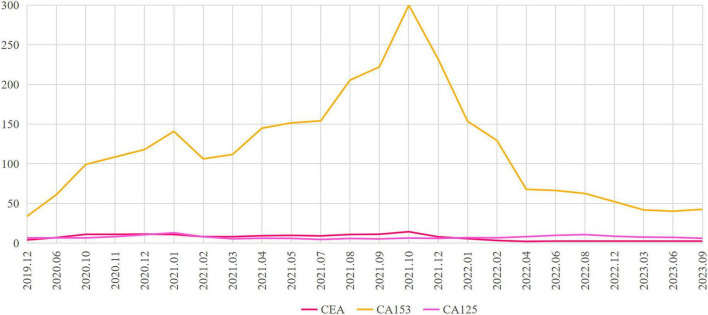
Serial CA 15-3 trend from December 2019 to September 2023. A gradual rise in CA 15-3 levels is seen during disease progression, followed by a rapid, sustained decline after initiation of abemaciclib.

Because the metastatic disease was confined to bone-only, purely osteolytic lesions, objective assessment of progression was inherently challenging under RECIST 1.1 (13, 14). Therefore, treatment decisions required integrated evaluation of imaging findings, clinical symptoms, and tumor marker dynamics (15, 16). In this case, follow-up imaging showed further expansion of the osteolytic lesions. Concurrently, serum CA15-3 levels continued to rise, and the patient reported worsening bone pain. Taken together, these findings were clinically suggestive of progression during palbociclib-based therapy, although unequivocal RECIST-defined progression could not be established. On this basis, and after integrated assessment of serial imaging changes, worsening bone pain, and tumor marker dynamics, the treating team decided to modify the treatment regimen. Accordingly, after abemaciclib was included in the National Health Insurance catalog in January 2022, treatment was switched to abemaciclib (150 mg bid) in combination with fulvestrant and zoledronic acid. The patient reported an overall improvement in quality of life following abemaciclib administration combined with endocrine therapy compared with her previous condition. The patient acknowledged the accessibility and convenience of the oral regimen while expressing concerns about adverse reactions such as diarrhea and fatigue. Regarding molecular testing, the patient declined further genetic testing for personal and financial reasons, but remained willing to undergo regular follow-up and imaging review to assess treatment efficacy. Progression-free survival (PFS) was defined as the time from initiation of abemaciclib plus fulvestrant therapy to documented radiologic progression, unequivocal clinical progression, death, or the last follow-up if no progression had occurred. After initiation of abemaciclib plus fulvestrant, serum CA15-3 levels declined steadily and remained within the normal range, with no evidence of radiologic progression through the last follow-up in September 2023 (Figure 2C). At that time, the patient remained progression-free. Figure 3 shows the changes in CA153 levels from December 2019 to September 2023.

## 3 Discussion

### 3.1 Mechanisms of acquired resistance

CDK4/6 inhibitors have emerged as promising therapies for treating patients with HR+/HER2- advanced breast cancer, both as first- and second-line treatment of advanced disease; the era of post-CDK4/6-inhibitor setting has started. However, drug resistance has remained a clinical challenge, with some patients showing primary resistance at the time of initial treatment, while others progressively developed acquired resistance over the course of treatment (17). The mechanisms of CDK4/6 inhibitor resistance are extremely complex and include abnormalities in multiple proteins and signaling pathways, such as *RB1* gene alterations, dysregulation of cell cycle proteins, FGFR pathway abnormalities, FAT1 deletion, TP53 pathway alterations, aurora kinase A (AURKA) upregulation, overactivation of the PI3K/AKT/mTOR pathway, CDK6 amplification, MYC upregulation, ESR1 mutations, ERBB2 activation and Cyclin E amplification (17, 18).

Based on their effects on the cell cycle, the mechanisms underlying resistance to CDK4/6 inhibitors can be classified into cell cycle-dependent and cell cycle-independent. Examples of cell cycle-dependent mechanisms include loss or mutation of the retinoblastoma protein (Rb), amplification of Cyclin-dependent kinase 2 (CDK2), and overexpression of CDK7 and CDK6, while cell cycle-independent mechanisms include overexpression of cell cycle upstream factors such as PI3K/AKT/mTOR and FGFR (19).

*RB1* loss is an established mechanism of CDK4/6 inhibitor resistance, demonstrated in cell lines and patient-derived xenograft models. In clinical practice, mutations or deletions in both *RB1 alleles* are seen in approximately 10% of patients with disease progression after CDK4/6 inhibitor therapy but not in patients receiving endocrine therapy alone (18). After *RB1* loss, cell cycle progression is driven in a CDK4/6-independent manner. AURKA plays an important role in cell division and is associated with both intrinsic and acquired resistance to CDK4/6 inhibitors and endocrine therapy (20). However, CDK6 amplification, seen in certain palbociclib-resistant cell lines after prolonged exposure to CDK4/6 inhibitors, promotes resistance; moreover, CDK6 knockdown restores sensitivity to CDK4/6 inhibitors (21). Overexpression of FGFR1 reduces sensitivity to CDK4/6 inhibitors by altering growth factor signaling pathways. Compensatory activation of CDK2 has been identified as an important mechanism contributing to CDK4/6 inhibitor resistance (18, 22–24). CDK2-selective inhibitors reversed the p53-deficient effect and restored cell cycle control, while wild-type TP53 was retained. Moreover, elevated CDK9 mRNA levels in plasma-derived exosomes correlate with palbociclib resistance (24).

### 3.2 Using ctDNA to guide therapy

Tumors undergo molecular changes during the treatment of advanced breast cancer. Monitoring ctDNA dynamics before and during treatment is an effective method to track these molecular changes. ctDNA monitoring has significant clinical value in CDK4/6 inhibitor therapy, specifically in early identification of drug resistance, personalized treatment guidance, dynamic assessment of efficacy, and monitoring of microscopic residual disease. Advances in technology have enabled widespread application of ctDNA screening, providing significant support for precision therapies. However, a major limitation of this case is the absence of molecular profiling, as the patient declined further genetic testing for personal and financial reasons. Therefore, alterations involving RB1, ESR1, PIK3CA, and the Cyclin E pathway were not assessed, and we cannot determine whether a specific molecular mechanism underlay the apparent benefit from abemaciclib after prior palbociclib exposure.

Several studies have explored treatment options for managing patients who progress after CDK4/6 inhibitor therapy. These include rechallenge with CDK4/6 inhibitors and the use of antibody-drug conjugates (ADCs). Although the major guidelines offer no standard recommendations for treating patients who develop resistance to CDK4/6 inhibitors, some therapeutic strategies are discussed below.

### 3.3 Sequential CDK4/6 inhibition: from palbociclib to abemaciclib

The MAINTAIN (ribociclib) and post-MONARCH (abemaciclib) studies have demonstrated that switching to another CDK4/6 inhibitor can still be of benefit in patients with HR+/HER2- advanced breast cancer. Therefore, the 2024 CSCO Guidelines have classified “cross-line CDK4/6” as a class II recommendation (25). In our patient, abemaciclib administered after clinical progression during palbociclib-based therapy was associated with progression-free survival exceeding 20 months. This observation suggests that prolonged disease control may be achievable in selected patients with bone-only HR+/HER2- metastatic disease, although the response assessment in this setting should be interpreted cautiously. The mechanism may involve the following aspects: first, the difference in selectivity between abemaciclib and palbociclib for CDK4 and CDK6. Although palbociclib has a comparable affinity for both CDK4 and CDK6, abemaciclib has a significantly higher affinity for CDK4 than CDK6 (18). Furthermore, CDK4 has a more prominent role than CDK6 in the development of hormone receptor-positive breast cancer (26). Second, abemaciclib has a broader spectrum of effects compared with palbociclib. In addition to CDK4 and CDK6, abemaciclib also acts on CDK2 and CDK9 (24, 27), which play an important role in the mechanisms of drug resistance in breast cancer cases. Third, standard adult dose of palbociclib is 125mg daily, administered orally for 21 consecutive days followed by a 7-day break. Abemaciclib is administered continuously at 150 mg twice daily. Palbociclib dosage might influence sustained pathway inhibition. These pharmacologic differences may partly explain the observed clinical benefit in this case; however, in the absence of molecular profiling, any mechanistic interpretation remains speculative.

A recent report (PMID: 39633462) (28) also underscored the clinical relevance of CDK4/6 inhibitor-based therapy in advanced luminal breast cancer with extensive bone/bone marrow involvement. Although that report does not directly establish the efficacy of sequential abemaciclib after palbociclib, it provides additional clinical context for CDK4/6 inhibitor use in heavily bone-involved disease. Taken together with emerging clinical trial data, our case adds to the ongoing discussion regarding sequential CDK4/6 inhibition in HR+/HER2− metastatic breast cancer.

### 3.4 New options for endocrine therapy: oral SERD

*ESR1* mutations are key to endocrine resistance. Oral SERD elacestrant is Food and Drug-approved as more effective than fulvestrant in patients with *ESR1* mutations who progress on CDK4/6 inhibitors. It is also less painful than intramuscular injections and may be considered for subsequent endocrine regimens in patients with *ESR1* mutations.

### 3.5 Targeted therapy plus endocrine combinations

*PIK3CA* mutations occur in approximately 40 % of tumors that progress on CDK4/6 inhibitors. Based on the SOLAR-1 and BYLieve trials, alpelisib combined with endocrine therapy is recommended as second-line treatment for managing these patients. Capivasertib, validated in the CAPItello-291 study, has been approved for tumors harboring PIK3CA, AKT1, or PTEN alterations (29). Everolimus requires no molecular testing; real-world data show a median PFS of approximately 5 months after progression on CDK4/6-inhibitors.

### 3.6 Replacement for chemotherapy

In China, conventional chemotherapy remains an important option after cancer progression on CDK4/6 inhibitors because it is widely available, well established, and supported by extensive clinical experience. Chemotherapy is indicated when disease progresses rapidly, a visceral crisis develops, or the response to prior endocrine or CDK4/6-inhibitor therapy is poor. Under these circumstances, endocrine-based regimens should be promptly replaced with cytotoxic chemotherapy.

### 3.7 Switching to ADCs

In recent years, ADCs have played an important role in the era of post-CDK4/6 inhibitors. ADCs are composed of monoclonal antibodies targeting specific antigens and small-molecule cytotoxic drugs linked by a linker that combines the potent lethal effects of traditional small-molecule chemotherapy with the tumor-targeting properties of antibody drugs. The DESTINY-Breast 04/06 study showed that, compared with physician-selected chemotherapy regimens, T-DXd treatment resulted in a significant improvement in PFS in HR+ patients with low/ultra-low HER2 expression. The TROPiCS-02 study showed that an ADC targeting TROP-2, sacituzumab govitecan, resulted in a significant improvement in PFS compared with physician-selected chemotherapy regimens in patients who had previously received CDK4/6 inhibitors. Based on the findings of these trials, both T-DXd and sacituzumab govitecan have emerged as clinically significant options after the progression on CDK4/6 inhibitor therapy in patients with HR+/HER2-advanced breast cancer.

## 4 Conclusion

This case suggests that sequential CDK4/6 inhibition with abemaciclib plus endocrine therapy may provide prolonged disease control in a patient with HR+/HER2- breast cancer and bone-confined metastasis after clinical progression during prior palbociclib-based therapy. However, because unequivocal RECIST-defined progression during prior therapy could not be established and molecular profiling was unavailable, this observation should be regarded as hypothesis-generating. Dynamic monitoring of CA15-3 may provide supportive information during disease assessment, but it must always be interpreted in conjunction with clinical symptoms and imaging findings. Sequential use of a different CDK4/6 inhibitor may be feasible in selected patients. However, larger prospective studies and biomarker-guided patient selection are needed to further evaluate this strategy.

## Data Availability

The original contributions presented in the study are included in the article/supplementary material, further inquiries can be directed to the corresponding author.
